# Predicting short stay total hip arthroplasty by use of the timed up and go-test

**DOI:** 10.1186/s12891-021-04240-6

**Published:** 2021-04-16

**Authors:** Ellen Oosting, Paul J. C. Kapitein, Suzan V. de Vries, Ellen Breedveld

**Affiliations:** 1 Department of Orthopedics, Hospital Gelderse Vallei, Willy Brandtlaan, 10 6716 RP Ede, the Netherlands; 2 Department of Physiotherapy, Hospital Gelderse Vallei, Ede, the Netherlands

**Keywords:** Total hip arthroplasty, Physical functioning, Risk stratification

## Abstract

**Background:**

One of the most important steps before implementing short stay total hip arthroplasty (THA) is establishing patient criteria. Most existing criteria are mainly based on medical condition, but as physical functioning is associated with outcome after THA, we aim to evaluate the added value of a measure of physical functioning to predict short-stay THA.

**Methods:**

We used retrospective data of 1559 patients who underwent an anterior THA procedure. Logistic regression analyses were performed to study the predictive value of preoperative variables among which preoperative physical functioning by use of the Timed Up and Go test (TUG) for short stay THA (< 36 h). The receiver operating characteristic (ROC) curve and Youden Index were used to define a cutoff point for TUG associated with short stay THA.

**Results:**

TUG was significantly associated with LOS (OR 0.84, 95%CI 0.82–0.87) as analyzed by univariate regression analysis. In multivariate regression, a model with the TUG had a better performance with an AUC of 0.77 (95%CI 0.74–0.79) and a R^2^ of 0.27 compared to the basic model (AUC 0.75, 95%CI 0.73–0.77, R^2^ 0.24). Patients with a preoperative TUG less than 9.7 s had an OR of 4.01 (95%CI 3.19–5.05) of being discharged within 36 h.

**Conclusions:**

Performance based physical functioning, measured by the TUG, is associated with short stay THA. This knowledge will help in the decision-making process for the planning and expectations in short stay THA protocols with the advantage that the TUG is a simple and fast instrument to be carried out.

## Introduction

Total hip arthroplasty (THA) is one of the most common and most successful orthopedic procedures [1]. With the aging population, the demand for THA and its economic burden are expected to grow considerably in the next decade [2, 3]. In the past, the length of stay (LOS) following primary THA has been 1 week or more; however, advances in surgical techniques and clinical pathways enabled faster recovery and shorter LOS [4]. These advances in (minimal invasive) surgical techniques, combined with multimodal analgesia and early rehabilitation have reduced LOS to an average of 2 to 4 days, even allowing for same-day discharge in selected patients [5]. Same-day THA can be safe and effective in eligible patients and may result in substantial cost savings [6, 7]. Furthermore, a shorter LOS may also improve patient satisfaction, allowing patients to recover in their private, comfortable home environment and achieve independence as early as possible [8]. Establishing inclusion criteria is one of the most important steps before implementing short stay or even same day THA [9]. Several studies concluded that patients with specific medical conditions, a low American Society of Anesthesiologists (ASA) classification (<III), undergoing primary arthroplasty, age < 75 and with support at home are eligible candidates for short stay joint arthroplasty [9–13]. While most studies have focused on demographic and medical factors, preoperative physical functioning has rarely been reported as a selection criterion. Although Bodrogi et al. defined worse physical functioning (Timed Up and Go-test, TUG> 10 s) as a relative exclusion criterion for same-day discharge after total joint arthroplasty (TJA), this recommendation was not based on an outpatient TJA population [14–16]. As other studies found that measurements of physical functioning were of relevance to predict recovery of functioning and LOS after total joint arthroplasty (TJA) [17–19], there are reasons to expect that preoperative physical functioning might also be an important predictor for short-stay THA with added value compared to the usual selection criteria.

In this study we evaluated the association between preoperative performance based physical functioning (the Timed Up and Go-test, TUG), for short stay elective THA (discharge within 36 h after surgery).

## Methods

We used data of a retrospective cohort of patients who underwent primary THA between 2015 and 2017 in the Gelderse Vallei Hospital in the Netherlands. We used data from all patients who had elective primary total hip arthroplasty by the anterior approach. Patient who underwent THA by the posterior approach were excluded as the posterior approach was only used in this hospital for specific cases like revision surgery. Patients who underwent bilateral THA were also excluded. Six orthopedic surgeons performed anterior THA surgery. Use of clinical data was approved by the local medical ethics committee of the Gelderse Vallei Hospital (BCWO 1804–076). This study complied with the principles in the Declaration of Helsinki. Data was extracted from the medical files by a data-specialist and checked, complemented and anonymized by the researcher. The STROBE guideline was used when drafting the manuscript.

In our pathway called “Active Recovery”, all patients had a preoperative screening by an anesthetist, a nurse and a physical therapist, including screening of physical functioning. Besides a screening of health conditions and comorbidities, preoperative physical fitness was evaluated by self-reported questionnaires and by performance assessment based to determine the risk for delayed recovery or complications. Moreover, patients were informed and advised about the importance of staying physically active and fit before surgery by the physical therapist. Expectations, goals, home situation and (necessary) help were discussed. Furthermore, patients were trained to walk with crutches. Patients were informed that a hospital stay of 1 or 2 nights was common. All patients had the same perioperative protocol. Both local and general anesthesia were used based on the indication of the anesthetist and preference of the patient.

Postoperatively, patients started to mobilize 4 h after surgery and were discharged when they were able to ambulate independently (with crutches or other walking aid), had no medical or wound problems and had sufficient help and care at home. Same day discharge was not possible yet due to logistics and organization of care.

### Outcome measures

The dependent variable LOS was dichotomized. Short stay THA was defined as a LOS less than 36 h (one overnight stay). Hours were counted from the time of surgery until discharge.

The independent variable was Preoperative physical functioning measured by TUG. The TUG test measures the domain of functional mobility and is recommended by The Osteoarthritis Research Society International (OARSI) [20]. Participants were asked to rise from a chair, walk three meters, turn, return and sit down, all as fast as possible. A lower score (in seconds) reflects better functional mobility. Use of a walking aid was permitted, but only when they also depended on a walking aid at home.

Other descriptive variables were:

- Preoperative: sex, body mass index (BMI, kg/m^2^), social status (living together, living alone with help from someone nearby or living alone), HOOS-PS (Hip disability and Osteoarthritis Outcome Score - Physical Function Short Form) [18] and the ASA classification [21]. As we only had a few patients with ASA score 4, ASA class 3 and 4 were merged as one category.
- Peroperative: anesthesia (general or spinal)
- Postoperative: discharge destination (home or rehabilitation)

Assuming at least 10 events per variable, the database is large enough for sufficient statistical power [22]. We used complete case analysis and checked for bias by evaluating if the missing data were related to baseline characteristics and ASA score (by Chi-square statistics) and age (by paired t-test).

Standard statistics were used for descriptive data. The variance inflation factor (VIF, cutoff 10) and the correlation matrix (cutoff 0.8) were used to test for multicollinearity [22].

First univariate regression was done for age, sex, ASA score, HOOS-PS, anesthesia and TUG. The statically significant variables age, sex and ASA score were used for multivariate logistic regression analysis. We evaluated 2 models: Firstly, a basic model with age, sex and ASA-score as variables. Secondly, we evaluated the basic model + TUG, to evaluate the added value of TUG. Goodness of fit was tested with the Hosmer & Lemeshow test and Nagelkerke R^2^ statistics. Receiver operating characteristic (ROC) curves were constructed with the logistic regression model to assess their predictive value using the area under the curve (AUC).

Furthermore, we performed a logistic regression with TUG as a dichotomous variable to be able to compare the results with other studies and to translate them into clinical practice. The optimal cutoff value was defined as the point on the ROC curve where Youden’s index (sensitivity+specificity− 1) was the highest. Sensitivity, specificity, positive and negative likelihood ratio were calculated based on the ROC curve.

SPSS Statistics 25 (SPSS Inc., Chicago, IL) was used for all statistical analyses.

## Results

A total of 1608 patients underwent a THA in the study period and 49 cases were excluded (revision surgery, posterolateral approach, simultaneous bilateral THA). All 1559 patients who were included underwent primary THA by anterior approach. Table 1 shows baseline characteristics. Mean BMI was 27.2 (SD 4.8) kg/m^2^ and 69% of all patients were female (Table 1). Mean TUG score was 10.8 s (SD 5.8).

**Table 1:** Characteristics of the study population

		Total	Fast recovery LOS 1 night	Normal recovery LOS ≥2 nights
			(< 36 h)	(≥36)
		*n* = 1559	*n* = 631	*n* = 928
**Preoperative**				
Age	mean (SD)	69.8 (9.3)	66.1 (8.3)	72.4 (9.1)
Sex	% women	68.8	55.9	77.6
BMI (kg/m2)	mean (SD)	27.2 (4.5)	26.9 (4.2)	27.4 (4.7)
Social status				
Living together	%	68.5	84.3	57.7
Help nearby	%	10.7	7.7	12.7
Living alone	%	20.8	7.9	29.5
ASA score				
I	%	25.0	35.7	17.8
II	%	59.8	57.3	61.6
II/IV	%	15.2	7.0	20.6
TUG (sec)	mean (SD)	10.8 (5.8)	8.8 (4.2)	12.1 (6.4)
HOOS-PS	mean (SD)	44.5 (17.3)	41.3 (16.5)	46.7 (17.6)
**Peroperative**				
Anesthesia				
General	%	53.9	54.5	53.4
Spinal	%	46.1	45.5	46.6
**Postoperative**				
LOS	mean (SD)	2.0 (1.3)		
Discharge location				
Home	%	89.0	98.1	82.8
Rehabilitation	%	11.0	1.9	17.2

*LOS* Length of hospital stay, *BMI* Body mass index, *ASA* American Society of Anesthesiologists classification, *TUG* Timed up and go test, *HOOS-PS* The Hip disability and Osteoarthritis Outcome Score - Physical Function Short Form, *SD* Standard deviation

Forty percent (*n* = 631) of all patients went home 1 day after surgery within 36 h, of whom 16% went home within 24 h (after 1 night). A total of 11% of all patients needed inpatient rehabilitation after discharge.

The ASA-score had two missing values and TUG had 44 missing values. In 28 cases patients did not attend preoperative physical therapy screening and in 16 cases the TUG was not executed.

There were no differences in age, sex or ASA-score between the dataset with and without missing data of TUG.

There was no multicollinearity as the VIF’s were all below 2 and all correlation coefficients were below 0.5, so the cutoff points were not reached.

Univariate regressions confirmed that sex (male), lower age, lower ASA-score, social status and faster time on the TUG were all independently associated with short stay LOS (Table 2.). HOOS-PS and anesthesia were not associated with LOS.

**Table 2:** Univariate association between baseline characteristics and short stay (< 36 h) THA

	OR (95%CI)	*p*
Age	0.92 (0.91–0.93)	0.000
Sex (male)	2.70 (2.17–3.37)	0.000
ASA score		
I	reference	
II	0.46 (0.37–0.59)	0.000
III/IV	0.17 (0.12–0.25)	0.000
TUG	0.84 (0.81–0.87)	0.000
HOOS-PS	1.00 (1.00–1.00)	0.382
BMI	0.98 (0.95–1.00)	0.058
Anesthesia (spinal)	0.95 (0.77–1.16)	0.592
Social status		
Living together	reference	
Help nearby	0.42 (0.28–0.63)	0.000
Living alone	0.19 (.013–0.27)	0.000

*ASA* American Society of Anesthesiologists classification, *TUG* Timed up and go test, *CI* Confidence interval

Table 3 shows the results of multivariate regression. A basic model with age, sex and ASA-score had an AUC of 0.75 (95% CI 0.73–0.78). TUG (OR 0.92, 95%CI 0.89–0.95) contributed significantly to basic model. The model with the TUG had the best performance with an AUC of 0.77 (95%CI 0.74–0.79) and R^2^ of 0.27.

**Table 3:** Multivariate association between baseline characteristics and short stay (< 36 h) THA

	MODEL 1 Basic variables OR (95%CI)	*p*	MODEL 2 Basic variables + TUG OR (95%CI)	*p*
Age	0.93 (0.92–0.94)	0.000	0.94 (0.93–0.95)	0.000
Sex (male)	2.81 (2.21–3.56)*	0.000	2.57 (2.00–3.30)	0.000
ASA score				
I	reference		reference	
II	0.56 (0.43–0.73)	0.000	0.63 (0.48–0.82)	0.001
III/IV	0.22 (0.14–0.33)	0.000	0.28 (0.18–0.44)	0.000
TUG	X		0.92 (0.89–0.95)	0.000
Nagelkerke’s R^2^	0.24		0.27	
Hosmer & Lemeshow test, *p*-value	0.26		0.30	
AUC	0.75 (0.73–0.78)		0.77 (0.75–0.79)	

*p < 0.05, *ASA* American Society of Anesthesiologists classification, *TUG* Timed up and go test, *OR* Odds ratio, *CI* Confidence interval, *AUC* Area under the curve

**Fig. 1:**
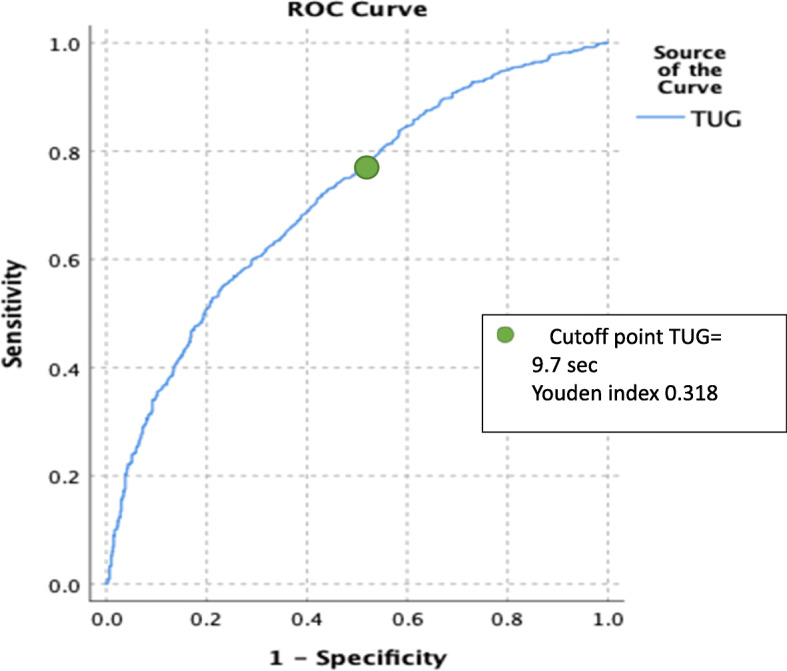
ROC curve of TUG with short stay THA

The cutoff point for the TUG was 9.7 s (Youden index 0.318, Fig. 1). Sensitivity was 79% and specificity 48%. Positive likelihood ratio 1.51 and negative likelihood 0.44. Patients with a TUG score less than 9.7 s had an OR of 4.01 (95%CI 3.19–5.05) to be discharged within 36 h.

## Discussion

In our retrospective cohort we aimed to evaluate the predictive value of preoperative physical functioning for short stay THA. Our results indicate that perioperative performance based physical functioning (TUG) was independently associated with short stay THA. A basic model (age, sex and ASA) with TUG, score had a slightly better predictive value compared to the basic model without TUG, with an acceptable AUC of 0.77. Adding the TUG to the existing screening models can be of added value in selecting eligible patients for short stay THA.

Like other studies, this study again confirms that the TUG is a predictor of LOS. The added value of the current study is that we used a cohort with unselected patients in a fast-track pathway approaching the outpatient setting. Several studies confirmed performance based measures like TUG or gait speed as independent determinants of LOS or functional recovery [15, 17, 19, 23, 24]. However, the focus in these studies was on predicting prolonged LOS or functional recovery of patients in a pathway with a mean LOS of 3–4 days. Holm et al. did study the role of preoperative TUG in discharge readiness for THA in a comparable fast track pathway as the current study, but did not find a relation in a relative small cohort of THA patients (*n* = 75). Although Bodrogi et al. stated in their review about management of patients undergoing same-day discharge primary total hip and knee arthroplasty that a Timed Up and Go Test > 10 s is a relative exclusion criteria for outpatient THA [14], TUG has not been used in selection criteria for outpatient THA [9, 10, 12, 13]. The next step is to evaluate the value of TUG as a predictor of outpatient THA.

Similar to the cutoff point that Bedrogi et al. mentioned in their review, we found a rounded cutoff point of 10 s. Several studies also calculated a cutoff point for TUG in relation to LOS or functional recovery after THA. Poitras et al. describe an association between preoperative TUG (cut off point 11.7 s) and LOS (cut off point 3 days) and a cutoff point of 10 s for the TUG for functional recovery 2 weeks after surgery. Elings et al. describe a cutoff point of 12.5 s [19] and Oosting et al. found a cutoff point of 10.5 s to predict delayed recovery of functioning (of respectively more than four and 3 days) [19]. In our study, patients with a TUG less than 9.7 s had an OR of 4.01 of being discharged within 36 h. Although TUG was of added value to a basic prediction model, the performance of the TUG score as a dichotomous variable on its own as prognostic test was moderate, so a low TUG score should not be used as an absolute exclusion criterion and should be part of a prognostic model including at least age, sex and comorbidity. Furthermore, the cutoff point of a test depends on the local pathway and context, so it should be validated in each local setting.

Most short stay (or outpatient) protocols primarily focus on ASA-score or other tools assessing medical condition like the recently developed Outpatient Arthroplasty Risk Assessment (OARA) [13]. This screening instrument has nine medical items to predict safe outpatient TJA and is effective for identifying patients who can safely undergo outpatient total joint arthroplasty. However, this is a one-dimensional approach and does not take into account the functional capabilities of patients. As reported in the study of Gromov et al., lack of safe mobilization might be one of the most common reasons for THA patients not being discharged at the day of surgery [25]. Therefore, it makes sense that better preoperative functional mobility is related to successful short stay THA. A measure of performance based physical functioning cannot simply be replaced by a questionnaire [16]. Our study found that the HOOS-PS was not associated with short-stay THA. Performance-based measures assess what an individual can do rather than what the individual perceives they can do. Furthermore, patients could under- or overestimate their functional ability by use of self-reported measures [26]. In our study both ASA and TUG were associated with short-stay THA, so we propose to take into account both physical functioning and comorbidity, by use of the ASA or the OARA score, in preoperative risk stratification to estimate whether a quick and uncomplicated recovery is likely.

Although social status is also related to LOS after THA (84% of the patients in the short stay group were living together vs 58% in the long-stay group) we did not include this variable in the regression model. In our experience, single patients who are motivated, confident and who have sufficient care and someone at home for the first days after discharge, are also candidates for short-stay THA. Horne et al. described this as a ‘Joint Coach’ in their enhanced recovery pathway [27].

This study had several strengths. We used a large cohort with patients without selecting candidates for short-stay THA prior to surgery, providing a good reflection of daily practice without being biased. We assume a large number of patients in our cohort who were discharged the next postoperative day are candidate for outpatient THA, provided that attention is paid to managing expectations, optimizing the mindset of patient and caregivers within a multidisciplinary approach and evidence based fast track protocols [5, 6, 14]. The strength of TUG as an objective measure for physical functioning lies in its wide use and simplicity of performance. TUG can be performed during preoperative physical therapy, which is part of most outpatient protocols [6] or even at the patients’ home. Furthermore, TUG is not only useful in predicting LOS after THA but may also be useful to predict long term outcome and other postoperative risks considering that TUG is also found to be associated with functional independence and risk of falling and frailty in elderly [28, 29] and with deep venous thrombosis after THA [30]. In addition, including preoperative measurement of physical functioning like TUG may be an important starting point to a more function tailored pathway. Van der Sluis et al. studied a function tailored approach and were able to reduce LOS by use of measurements of physical functioning, reduction of inactivity and stimulation of self-efficacy of the patients [31]. Functional mobility, measured by TUG, is a modifiable risk factor and could be a target in preoperative preparation of patients.

This study has several limitations. First, it was a single-center retrospective study without external validation. Second, although we took into account confounding factors like age, sex and ASA score, there are more preoperative factors related to short-stay THA, which may result in some residual bias. Thirdly, we only evaluated one single test of physical functioning. Further studies are necessary to validate the use of TUG or other measures of physical functioning in preoperative risk stratification for short-stay or outpatient THA and their added value to other existing risk assessment instruments like the OARA score.

## Conclusions

Patients with a better performance based physical functioning, as before measured by the TUG, are more likely to have a short stay after THA. This knowledge can help in the decision-making process for the planning and expectations after THA with the advantage that the TUG is a simple and fast instrument to be carried out. Since TUG had added value to predict short stay THA in a basic prediction model with ASA-score, age and sex, it could be a valuable patient selection criterium for outpatient THA. Further studies are necessary to validate the use of the TUG in preoperative prediction models for outpatient THA.

## Data Availability

The datasets used and/or analyzed during the current study are available from the corresponding author on reasonable request.

## Abbreviations

THA: Total Hip Arthroplasty
ASA: American Society of Anesthesiologists
HOOS-PS: Hip disability and Osteoarthritis Outcome Score - Physical Function Short Form
LOS: Length of Stay
OARSI: Osteoarthritis Research Society International
BMI: Body Mass Index
VIF: Variance Inflation Factor
OR: Odds Ratio
CI: Confidence Interval
ROC: Receiver Operating Characteristic
SD: Standard Deviation
AUC: Area Under the Curve
